# Willingness of US Men Who Have Sex with Men (MSM) to Participate in Couples HIV Voluntary Counseling and Testing (CVCT)

**DOI:** 10.1371/journal.pone.0042953

**Published:** 2012-08-14

**Authors:** Bradley H. Wagenaar, Lauren Christiansen-Lindquist, Christine Khosropour, Laura F. Salazar, Nanette Benbow, Nik Prachand, R. Craig Sineath, Rob Stephenson, Patrick S. Sullivan

**Affiliations:** 1 Department of Epidemiology, Rollins School of Public Health, Emory University, Atlanta, Georgia, United States of America; 2 Department of Health Promotion and Behavior, Institute of Public Health, Georgia State University, Atlanta, Georgia, United States of America; 3 Chicago Department of Public Health, Chicago, Illinois, United States of America; 4 Hubert Department of Global Health, Rollins School of Public Health, Emory University, Atlanta, Georgia, United States of America; Yale School of Public Health, United States of America

## Abstract

**Background:**

We evaluated willingness to participate in CVCT and associated factors among MSM in the United States.

**Methods:**

5,980 MSM in the US, recruited through MySpace.com, completed an online survey March-April, 2009. A multivariable logistic regression model was built using being “willing” or “unwilling” to participate in CVCT in the next 12 months as the outcome.

**Results:**

Overall, 81.5% of respondents expressed willingness to participate in CVCT in the next year. Factors positively associated with willingness were: being of non-Hispanic Black (adjusted odds ratio [aOR]: 1.5, 95% confidence interval [CI]: 1.2–1.8), Hispanic (aOR: 1.3, CI: 1.1–1.6), or other (aOR: 1.4, CI: 1.1–1.8) race/ethnicity compared to non-Hispanic White; being aged 18–24 (aOR: 2.5, CI: 1.7–3.8), 25–29 (aOR: 2.3, CI: 1.5–3.6), 30–34 (aOR: 1.9, CI: 1.2–3.1), and 35–45 (aOR: 2.3, CI: 1.4–3.7) years, all compared to those over 45 years of age; and having had a main male sex partner in the last 12 months (aOR: 1.9, CI: 1.6–2.2). Factors negatively associated with willingness were: not knowing most recent male sex partner’s HIV status (aOR: 0.81, CI: 0.69–0.95) compared to knowing that the partner was HIV-negative; having had 4–7 (aOR: 0.75, CI: 0.61–0.92) or >7 male sex partners in the last 12 months (aOR: 0.62, CI: 0.50–0.78) compared to 1 partner; and never testing for HIV (aOR: 0.38, CI: 0.31–0.46), having been tested over 12 months ago (aOR: 0.63, CI: 0.50–0.79), or not knowing when last HIV tested (aOR: 0.67, CI: 0.51–0.89), all compared to having tested 0–6 months previously.

**Conclusions:**

Young MSM, men of color, and those with main sex partners expressed a high level of willingness to participate in couples HIV counseling and testing with a male partner in the next year. Given this willingness, it is likely feasible to scale up and evaluate CVCT interventions for US MSM.

## Introduction

The number of new HIV diagnoses among men who have sex with men (MSM) has increased from an estimated 19,174 in 2005 to 29,341 in 2009, with MSM accounting for approximately 61% of all new HIV diagnoses in 2009 [1]. Despite the high proportion of HIV cases occurring among US MSM, the number of best-evidence interventions targeting MSM is low relative to their contribution to the US HIV epidemic [2]. Thus, there is an urgent need to identify and create evidence-based interventions and prevention services that are culturally appropriate for and acceptable to MSM.

An important avenue for addressing HIV prevention among MSM is considering prevention interventions and health services targeting levels beyond that of the individual. For example, a recent study conducted in five US cities estimated that 68% of HIV transmissions among MSM were attributable to main sex partners [3], which suggests that couples-based HIV interventions could be helpful in addressing the epidemic in this population. The proportion of transmission from a main sex partner was even higher among younger MSM, among whom rates of new HIV infections are rising most quickly [3].

Couples voluntary HIV counseling and testing (CVCT) has been used in Africa as an effective behavioral intervention to prevent HIV transmission among heterosexual couples [4], is supported programmatically by the US Government’s PEPFAR program in countries outside the United States as a prevention service, and is recommended for consideration in male couples in PEPFAR countries [5]. In CVCT, couples receive pretest information, counseling, and test results together and are counseled on ways to prevent HIV transmission within the relationship. Data from Africa suggest that providing HIV counseling and testing to a couple together is an effective intervention for reducing HIV transmission as compared to counseling the female partner alone [6], [7]. However, limited data are available on the feasibility of CVCT for MSM in the United States.

As part of a broader mixed-methods strategy to evaluate the acceptability of CVCT for US MSM, we collected and analyzed data from a large sample of internet-using MSM about willingness to receive HIV counseling and testing with a male partner and to identify factors associated with willingness to participate in CVCT. These data may help inform development of CVCT services to prevent HIV infections among MSM in the United States.

## Methods

MSM were recruited online through selective placement of banner advertisements displayed on a social networking website (MySpace.com) from March-April 2009. Methods have been previously reported [8]. Briefly, MySpace.com users in the United States who reported being male, 18 years of age or older, and gay, bisexual or unsure in their MySpace profile were exposed to banner advertisements. Participants who clicked through the banner advertisements were directed to an online eligibility screener. Eligible respondents were male, ≥18 years of age, and reported at least one male sex partner in the preceding 12 months. Eligible participants were shown the online informed consent module; those who consented were administered an internet-based survey.

Demographic information of interest included participants’ age, race/ethnicity, census region, and education. Questions pertaining to the participants’ behaviors used a 12 month recall period, and included: number of male sex partners, having a main male sex partner (one who they felt “committed to above all others”), having unprotected anal intercourse (UAI) with male sex partners, HIV testing history, and HIV status of their most recent male sex partner. Men who reported being diagnosed with HIV in the survey were excluded from our analyses due to potential differential motivations for participating in CVCT. Additionally, we retained only the first response when multiple responses were collected with a non-unique IP address.

Our primary outcome was self-reported willingness to participate in CVCT with a male sex partner in the next 12 months. Participants were first provided a description of CVCT as a service offered to male-female couples in Africa in which both members of a couple participate together in all phases of the HIV counseling and testing process. Participants were then asked: “If couples testing, where you and a sex partner got your HIV test results back together, were available in the United States, how likely is it that you would be tested together with a male sex partner in the next year?” Responses were collected on a 4-part Likert scale (definitely would, probably would, probably would not, definitely would not), with separate options for “don’t know”, and “prefer not to answer.”

Initial attempts to analyze results using ordinal logistic regression, with willingness as a 4-staged outcome, indicated that the proportional odds assumption was violated. Therefore, we dichotomized willingness outcomes into “willing” (participants who responded that they “would definitely” or “would probably” get tested with a partner in the next 12 months) and “unwilling” (participants who responded that they “probably would not” or “definitely would not” get tested with a partner in the next 12 months).

Participants were asked to indicate one or more reasons why they responded that they were willing or not willing to participate in CVCT from a pre-specified list constructed based on our earlier qualitative work [9]. Men who indicated multiple reasons for being willing or unwilling were then asked to choose the main reason for their willingness or unwillingness to participate in CVCT.

Statistical analyses were performed using SAS 9.3 (Cary, NC) and associations were evaluated for statistical significance at α  = 0.05 using two-tailed tests. Bivariate analyses were conducted to determine whether demographic and behavioral factors were associated with willingness to participate in CVCT with a male sex partner in the next 12 months. Number of male sex partners in the last 12 months and age were not linearly associated with willingness to participate in CVCT by estimated logit plots, and were therefore categorized and entered into the models as categorical variables. Variables considered for inclusion were: race/ethnicity, age, having had a main male sex partner in last 12 months, most recent male sex partner’s HIV status, having had unprotected anal intercourse in last 12 months, number of male sex partners in last 12 months, and time since last HIV test. Variables that were bivariately associated with willingness at p<0.2 were then entered into a multivariate logistic regression model. A backward elimination strategy (with SLSTAY  = .05) was used to identify factors significantly associated with willingness to participate in CVCT. Once a final first-order model was achieved, we conducted backward elimination of all two-way interactions of the remaining first-order variables to identify statistically important interactions of first-order covariates.

Because men in committed relationships may be more likely to utilize CVCT, we conducted a secondary analysis restricted to men who reported having a main sex partner in the last 12 months. The same analytical procedures described above were conducted to determine if factors associated were similar or different. If a restricted model adjusted odds ratio was within10% of the combined model estimate it was considered not to be meaningfully different.

Wald chi-square tests were used to establish significance of individual predictors, whereas likelihood ratio tests were used to evaluate significance of groups of predictors. Collinearity between predictors was assessed at each variable elimination step using variance inflation factors, variance decomposition proportions, and condition indices [10]. Hosmer and Lemeshow’s goodness of fit test and the area under the receiver operating curve were used to evaluate fit and discrimination of the final model, respectively.

## Results

Overall, 8,257,271 MySpace advertising impressions resulted in 30,559 survey click-throughs over a 29-day period. 17,254 (54% of click-throughs) completed the questions used to determine eligibility, 11,714 (68% of respondents to eligibility questions) were eligible to participate, and 9,005 (77% of eligible respondents) consented to participate in the study. We excluded 240 respondents who reported being HIV positive, and 138 response profiles with a non-unique IP address, retaining the first survey response from duplicate IP addresses. Of the remaining respondents, 5,362 (62%) responded to the question pertaining to willingness to participate in CVCT in the next 12 months with a male sex partner, and were included in final analyses. Compared to participants who completed the survey and were included in our analysis, those who consented but did not answer questions pertaining to participation in CVCT were more likely to be non-Hispanic black or of unknown race and were less likely to have had UAI with a male partner in the last 12 months.

A summary of demographic and behavioral characteristics of participants included in these analyses are presented in Table 1. Over two-thirds of participants were between the ages of 18 and 24, and most reported having at least one main male sex partner in the last 12 months. Slightly less than two-thirds of respondents reported having engaged in UAI in the last 12 months. Nearly half had received an HIV test in the last 12 months, while about a quarter had never been tested for HIV.

**Table 1:** Demographic and behavioral characteristics of 5,980 US men who have sex with men (MSM) who responded to Online Health and Technology Survey, 2009.

Characteristic	n (%) unless noted
**Race/Ethnicity**	
Non-Hispanic White	2,582 (43.2)
Non-Hispanic Black	778 (13.0)
Hispanic	1,897 (31.7)
Other*	664 (11.1)
Unknown	59 (1.0)
**Age**	**21 (median), 7 (IQR)**
18–24	4,083 (68.3)
25–29	957 (16.0)
30–34	401 (6.7)
35–45	404 (6.8)
Over 45	135 (2.3)
**Education**	
College, post graduate, or professional school	918 (15.4)
Some college, associate’s or technical degree	2,557 (42.8)
High school or GED	2,017 (33.7)
Less than high school	421 (7.0)
Don’t know/prefer not to answer	67 (1.1)
**Region**	
Midwest	924 (15.5)
North	787 (13.2)
South	2,137 (35.7)
West	1,846 (30.9)
Missing	286 (4.8)
**Had a main male sex partner in last 12 months**	
Yes	4,366 (73.0)
No	1,610 (26.9)
Missing	4 (0.07)
**Most recent male sex partner’s HIV status**	
Negative	4,183 (70.0)
Positive	108 (1.8)
Unknown	1,689 (28.2)
**Had unprotected anal intercourse in last 12 months**	
Yes	3,851 (64.4)
No	2,123 (35.5)
Missing	6 (0.10)
**Number of male sex partners in last 12 months**	
1	1,468 (24.5)
2–3	1,737 (29.0)
4–7	1,511 (25.3)
>7	1,259 (21.1)
Missing	5 (0.08)
**Time since last HIV test**	
0–6 months	1,938 (32.4)
7–12 months	894 (15.0)
>12 months	959 (16.0)
Never tested	1,659 (27.7)
Unknown	530 (8.9)
**Willingness to participate in CVCT**	
Definitely would	3,016 (50.4)
Probably would	1,354 (22.6)
Probably would not	659 (11.0)
Definitely would not	333 (5.6)
Prefer not to answer	49 (0.82)
Don’t know	569 (9.5)

* Includes Asian/Pacific Islander (n = 137), Native American/Alaska Native (n = 115), Multi-racial (n = 313), “other” (n = 99).

Excluding 49 participants who responded “don’t know” and 569 participants who responded “prefer not to answer” to the CVCT willingness question, 81.5% (4,370) of MSM were willing to participate in CVCT in the next 12 months, with 56.2% (3,016) responding they would definitely participate, 25.3% (1,354) responding they probably would participate, 12.3% (659) responding they probably would not participate, and 6.2% (333) responding they definitely would not participate (Table 2). Also excluding non-respondents to CVCT questions, of 1,421 men responding that they had never tested for HIV, 72.1% (1,025) stated they would be willing to participate in CVCT in the next 12 months (Table 2).

**Table 2:** Demographic and behavioral characteristics associated with willingness§ to participate in CVCT with a male sex partner in the next year among US men who have sex with men (MSM) who responded to Online Health and Technology Survey, 2009.

Characteristic	Willing§ n (%)	Unwilling¶ n (%)	Unadjusted OR N = 5,362** (95% CI)	Adjusted OR Combined N = 5,355†† (95% CI)	Adjusted OR Men with main sex partner only N = 3,965*** (95% CI)
**Total**	**4,370 (81.5)**	**992 (18.5)**			
**Race/Ethnicity** §§					
Non-Hispanic White	1,783 (78.5)	487 (21.5)	1.0 (reference)	1 (reference)	1 (reference)
Non-Hispanic Black	603 (84.5)	111 (15.5)	1.5 (1.2, 1.9)†	1.5 (1.2, 1.8)*	1.4 (1.0, 1.9)*
Hispanic	1,438 (83.0)	295 (17.0)	1.3 (1.1, 1.6)†	1.3 (1.1, 1.6)†	1.3 (1.1, 1.6)*
Other	503 (84.4)	93 (15.6)	1.5 (1.2, 1.9)*	1.4 (1.1, 1.8)*	1.6 (1.1, 2.2)*
Unknown	43 (87.8)	6 (12.2)	1.9 (0.8, 4.6)	2.0 (0.84, 4.9)	1.9 (0.65, 5.3)
**Age** §§					
18–24	3,019 (81.9)	668 (18.1)	2.4 (1.6, 3.6)†	2.5 (1.7, 3.8)†	2.8 (1.6, 4.9)†, ¶¶
25–29	714 (82.6)	150 (17.4)	2.5 (1.7, 3.9)†	2.3 (1.5, 3.6)†	2.7 (1.5, 4.8)†, ¶¶
30–34	273 (79.4)	71 (20.6)	2.1 (1.3, 3.3)*	1.9 (1.2, 3.1)*	2.0 (1.1, 3.8)*
35–45	289 (82.1)	63 (17.9)	2.4 (1.5, 3.9)†	2.3 (1.4, 3.7)†	2.3 (1.2, 4.4)*
Over 45	75 (65.2)	40 (34.8)	1.0 (reference)	1.0 (reference)	1.0 (reference)
**Education**					
College, post graduate, or professional school	677 (80.7)	162 (19.3)	1.0 (reference)	n.s	n.s
Some college, associate’s or technical degree	1,888 (81.6)	427 (18.4)	0.95 (0.77, 1.2)	n.s	n.s
High school or GED	1,463 (82.1)	318 (17.9)	0.91 (0.74, 1.1)	n.s	n.s
Less than high school	298 (81.0)	70 (19.0)	0.98 (0.72, 1.3)	n.s	n.s
Don’t know/prefer not to answer	44 (74.6)	15 (25.4)	1.4 (0.77, 2.6)	n.s	n.s
**Census region**					
Midwest	672 (82.4)	144 (17.6)	1.0 (reference)	n.s	n.s
North	566 (80.7)	135 (19.3)	0.90 (0.69, 1.2)	n.s	n.s
South	1,572 (81.7)	352 (18.3)	0.96 (0.77, 1.2)	n.s	n.s
West	1,352 (81.3)	312 (18.8)	0.93 (0.75, 1.2)	n.s	n.s
Missing	208 (80.9)	49 (19.1)			
**Had a main male sex partner in last 12 months**					
No	1,010 (72.4)	384 (27.6)	1.0 (reference)	1.0 (reference)	n/a
Yes	3,358 (84.7)	607 (15.3)	2.1 (1.8, 2.4)†	1.9 (1.6, 2.2)†	n/a
Missing	2 (66.7)	1 (33.3)			
**Most recent male sex partner’s HIV status** §§					
Negative	3,174 (83.7)	620 (16.3)	1.0 (reference)	1.0 (reference)	1.0 (reference)
Positive	78 (80.4)	19 (19.6)	0.80 (0.48, 1.3)	0.71 (0.42, 1.2)	0.60 (0.35, 1.1)¶¶
Unknown	1,118 (76.0)	353 (24.0)	0.62 (0.53, 0.72)†	0.81 (0.69, 0.95)*	0.79 (0.64, 0.97)*
**Had unprotected anal intercourse in last 12 months**					
No	1,509 (80.0)	378 (20.0)	1.0 (reference)	n.s	n.s
Yes	2,857 (82.3)	613 (17.7)	1.2 (1.0, 1.3)*	n.s	n.s
Missing	4 (0.80)	1 (0.2)			
**Number of male sex partners in last 12 months** §§					
1	1,067 (82.4)	228 (17.6)	1.0 (reference)	1.0 (reference)	1.0 (reference)
2–3	1,282 (82.4)	274 (17.6)	1.0 (0.82, 1.2)	0.89 (0.73, 1.1)	0.90 (0.70, 1.2)
4–7	1,113 (81.2)	258 (18.8)	0.92 (0.76, 1.1)	0.75 (0.61, 0.92)*	0.74 (0.57, 0.95)*
>7	905 (79.7)	231 (20.3)	0.84 (0.68, 1.0)	0.62 (0.50, 0.78)†	0.64 (0.49, 0.84)†
Missing	3 (75.0)	1 (25.0)			
**Time since last HIV test** §§					
0–6 months	1,571 (87.3)	228 (12.7)	1.0 (reference)	1.0 (reference)	1.0 (reference)
7–12 months	704 (85.5)	119 (14.5)	0.86 (0.68, 1.1)	0.86 (0.67, 1.1)	0.76 (0.57, 1.0)¶¶
>12 months	690 (80.7)	165 (19.3)	0.61 (0.49, 0.76)†	0.63 (0.50, 0.79)†	0.60 (0.46, 0.79)†
Never tested	1,025 (72.1)	396 (27.9)	0.38 (0.31, 0.45)†	0.38 (0.31, 0.46)†	0.38 (0.30, 0.48)†
Unknown	380 (81.9)	84 (18.1)	0.66 (0.50, 0.86)*	0.67 (0.51, 0.89)*	0.64 (0.45, 0.90)*

* P<.05 (Wald X^2^).

† P<.001 (Wald X^2^).

§ Includes 3,016 participants who indicated that they “definitely would”, and 1,354 participants who indicated that they “probably would”, participate in CVCT in the next 12 months with a male partner.

¶ Includes 659 participants who indicated that they “probably would not”, and 333 participants who indicated that they “definitely would not”, participate in CVCT in the next 12 months with a male partner.

** From 5,980 total respondents, excludes 569 men responding “don’t know” and 49 men responding “prefer not to answer” regarding their willingness to participate in CVCT in the next 12 months.

†† From 5,362 respondents to CVCT questions, excludes 3 men who were missing information on main sex partner in last 12 months and 4 men who were missing number of male sex partners in last 12 months.

§§ Construct p-values for adjusted model: 0.0003 (race/ethnicity), 0.0004 (age), <0.0001 (number of male sex partners), 0.021 (male sex partner HIV status), <0.0001 (Time since last HIV test).

¶¶ ‘Meaningfully’ different from combined model (over 10% different).

*** From 5,362 respondents to CVCT questions, includes only men who responded they had a main male sex partner in last 12 months.

n.s.  =  non-significant, eliminated during backward elimination for given model.

n/a  =  not applicable in given model.

No two-way interaction was retained in the final model. The final multivariable model achieved an area under the ROC curve of 0.66 and had no significant multicollinearity or lack-of-fit (Hosmer and Lemeshow’s p-value  = 0.96).

After adjusting for the other variables in the final multivariable model, race/ethnicity remained significantly associated with willingness to participate in CVCT, with men of non-Hispanic Black, Hispanic, and other race/ethnicity being significantly more likely to express willingness to participate in CVCT compared to non-Hispanic White men (Table 2). Compared to men aged over 45, men aged 18–24 years were 2.5 (CI: 1.7–3.8) times, men aged 25–29 years were 2.3 times (CI: 1.5–3.6), men aged 30–34 years were 1.9 times (CI: 1.2–3.1), and men aged 35–40 years were 2.3 times (CI: 1.4–3.7) more likely to express willingness to participate in CVCT in the next 12 months.

Men having at least one main male sex partner in the last 12 months were 1.9 (CI: 1.6–2.2) times more likely to be willing to participate in CVCT. In addition, men having 4–7 male sex partners in the last 12 months were 25% (CI: 8%-39%) less likely to be willing to participate in CVCT and men with 7+ male sex partners were 38% (CI: 22%-50%) less likely, both compared to men with one sex partner in the last 12 months. Men who did not know the HIV status of their last male sex partner were 19% (CI: 5%-31%) less likely to be willing to participate in CVCT in the next year. Finally, men who had never been HIV tested, did not know the time since their last test, and men who had been tested over 12 months previously, had significantly reduced odds of willingness to participate in CVCT, all compared to men who had been tested in the past 0–6 months (Table 2).

Restricting analyses to men reporting a male sex partner in the last 12 months did not change factors associated in a multivariable model of willingness to participate in CVCT. Yet, in this restricted model, men aged 18–24 or 25–29 years had ‘meaningfully’ higher odds of willingness compared to the combined model (Table 2). Also, men whose most recent male sex partner’s HIV status was positive, and men who had been tested 7–12 months previously had ‘meaningfully' lower odds of willingness, compared to the combined model.

Of the 4,370 men who reported that they were willing to participate in CVCT, 79.4% selected: “we would both know each other’s HIV status” as a reason for wanting to participate, and 24.3% selected this as their main reason for participation (Table 3). Other relationship factors cited as reasons why they were willing to get tested including: “to support each other” (75.2% endorsed, 16.5% main reason) and “would strengthen us as a couple” (68.7% endorsed, 11.4% main reason).

**Table 3:** Reasons and the most important reason for wanting to participate in CVCT with a male sex partner in the next year among 4,370* US men who have sex with men who reported willingness to test for HIV together with a partner, and who provided at least one reason for willingness, United States, 2009.

Reason	Any Reason n (%)	Most Important Reason n (%)
We would both know each other’s HIV status	3,470 (79.4)	1,060 (24.3)
To support each other	3,285 (75.2)	722 (16.5)
Would strengthen us as a couple	3,003 (68.7)	548 (11.4)
I would be confident that I knew his HIV status	2,902 (66.4)	330 (6.9)
To protect myself if my partner is positive	2,795 (64.0)	333 (7.0)
To protect my partner if I am positive	2,760 (63.2)	249 (5.2)
Would give us a chance to talk about rules for our relationship	2,222 (50.9)	107 (2.2)
It would help to have a counselor if one of us was positive	2,004 (45.9)	86 (1.8)
If we were both negative, we could stop using condoms	1,323 (30.3)	189 (3.9)
Some other reason	n/a	68 (1.4)

* Includes 3,016 participants who indicated that they “definitely would”, and 1,354 participants who indicated that they “probably would”, participate in CVCT in the next 12 months with a male partner.

Of the 992 men who reported that they were not willing to participate in CVCT, 55.4% selected “I would rather learn my own status first and then tell my partner” as a reason for not wanting to participate, and 22.5% selected this as their main reason for not wanting to participate (Table 4). Other common responses were: “I don’t have a regular male sex partner” (39.0% endorsed, 11.8% main reason), and “the counselor could ask me questions I wouldn’t want to answer with my partner there” (28.9% endorsed, 4.1% main reason).

**Table 4:** Reasons and the most important reason for not wanting to participate in CVCT with a male sex partner in the next year among 992* US men who have sex with men who responded reported being unwilling to test for HIV together with a partner, and who provided at least one reason for lack of willingness, United States, 2009.

Reason	Any Reason n (%)	Most Important Reason n (%)
I would rather learn my own status first and then tell my partner	550 (55.4)	223 (22.5)
I don’t have a regular male sex partner	387 (39.0)	117 (11.8)
The counselor could ask me questions I wouldn’t want to answer with my partner there	287 (28.9)	41 (4.1)
I’m afraid that I might be positive	178 (17.9)	53 (5.3)
It would be hard to schedule time together	169 (17.0)	25 (2.5)
I am not at risk for HIV	159 (16.0)	31 (3.1)
My partner is not at risk for HIV	152 (15.3)	13 (1.3)
I don’t need to be tested	142 (14.3)	26 (2.6)
I’m afraid that my partner might be positive	140 (14.1)	10 (1.0)
I am in a monogamous relationship	134 (13.5)	57 (5.7)
My partner would not want to be tested together even if I wanted to be tested together	113 (11.4)	20 (2.0)
I don’t want my partner to know my HIV status	57 (5.8)	4 (0.40)
I don’t want to know my partner’s HIV status	38 (3.8)	4 (0.40)
Some other reason	n/a	38 (3.8)

* Includes 659 participants who indicated that they “probably would not”, and 333 participants who indicated that they “definitely would not”, participate in CVCT in the next 12 months with a male partner.

## Discussion

Young, internet-using MSM expressed a high level of willingness to participate in couples counseling and testing for HIV with a male partner in the next 12 months: 81.5% of men reported that they would definitely or probably test with a male partner in the coming year, if such a service were available. Predictors of increased willingness were being of non-Hispanic Black, Hispanic, or “other” race/ethnicity, younger age, having at least one main sex partner in the last year, having fewer sex partners in the last year, knowing one’s most recent male sex partner to be HIV negative, and reporting having a recent HIV test.

These data are consistent with the results of our reported qualitative work, in which a high level of willingness to use CVCT was expressed by MSM in focus groups in Seattle, Atlanta, and Chicago [9]. The themes regarding motivation to use CVCT from those focus group discussions – e.g., expression of commitment to a relationship, desire to support their partner in the testing process, and as a means of assuring disclosure of HIV serostatus – were also reflected in reasons that men in our online survey reported for intention to use a couples counseling and testing service.

Our finding that younger men were more likely to be willing to participate in CVCT with a male partner than older men is encouraging, because we have previously estimated, and others have found in cohort studies, that HIV transmission from main partners may be especially prominent among younger MSM [3], [11]. This structured survey did not provide additional information about what underlies this difference in willingness by age; however, it is possible that there are generational differences in experiences and norms around HIV testing that lead younger men to be more open to different testing settings. These possibilities should be explored in subsequent qualitative work.

Men who had never tested for HIV, or for whom HIV testing was more distal, had significantly lower odds of willingness to participate in CVCT, compared to more recent testers. Even so, over 70% of those never tested for HIV expressed willingness to engage in CVCT in the next 12 months. This suggests that offering CVCT might attract MSM who haven’t historically tested to seek testing as part of a couple. As CVCT interventions are developed and brought to scale in the US, further mixed-methods research should investigate how reported willingness maps to use of the service and how to encourage those never HIV tested to utilize CVCT services.

Men who had a main male sex partner in the last year were more likely to be willing to participate in CVCT than men with only casual partners. Among those who were willing to participate, over two-thirds indicated that one reason for wanting to participate was to strengthen them as a couple. These data, together with results from qualitative work on willingness among MSM to use CVCT [9], suggest that men may be particularly willing to receive HIV testing as part of a more committed relationship. The reasons men would value the service were nearly equally divided between those related to addressing HIV risk with a partner, and those addressing strengthening the relationship and supporting one another. This is resonant with recent calls to consider HIV prevention approaches which promote the stability of male couples, as part of a comprehensive HIV prevention strategy for MSM [12].

Several of our findings suggest that men who did not express intention to test for HIV with a male partner may have had reservations which were more primarily related to HIV testing than to the idea of testing with a partner. For example, men who had not been tested for HIV recently were less likely to report willingness to participate in CVCT. Additionally, most of the reasons and main reasons participants cited for not wanting to participate in CVCT are consistent with reasons why persons do not test for HIV individually [13], [14], [15]. It is important to develop multiple testing approaches to increase testing frequency for MSM.

Another important concern expressed by those unwilling to use CVCT in the next 12 months was that the counselor would ask questions they would rather not answer in front of their partner. This reflects a misunderstanding of the CVCT process [16]. In traditional individual HIV counseling and testing, which most MSM in the US have likely experienced [17], individual risk inventory is a routine part of risk assessment and development of a prevention plan. CVCT, however, is future-focused, and CVCT counselors are trained to help couples develop an HIV prevention plan while avoiding historical risk inventories that might lead to conflict within the relationship or the session. This concern, however, is important in that it suggests that specific educational efforts will be required to reassure men considering CVCT that they will not be required to disclose their history of past sex partners in front of their partner.

The present study has important limitations. Participants included in these analyses are a convenience sample of users of a social networking site, and do not represent all MSM Myspace.com users, or any other group of MSM in the United States. Men who may have sex with men, but who did not disclose their sexual orientation as gay, bisexual, or unsure in their MySpace profile were not recruited by targeting of their online profiles; thus, these men are not represented in our sample. Since many of the questions in our survey were sensitive in nature, it is possible that some participants did not accurately report their risk behaviors, which could potentially subject our findings to social desirability bias [18]. However, this may not be as great of a concern since our survey was administered online, and participants are generally more honest about their risk behaviors when a computer-assisted survey method is used instead of traditional survey methods [19]. Last, hypothetical interest in using HIV prevention services, included testing services, may not reflect actual use of those services when they are eventually available and also relies on existing respondent knowledge of the testing process [20].

Our work also offers a number of strengths. Although not representative, our sample comes from an emerging, highly-inclusive public space. According to the Pew Research Institute, in 2011, 94% of Americans aged 18–29 used the internet, and over 80% of these individuals had a social networking account such as MySpace or Facebook [21], [22]. Second, our approach allowed us to accrue responses from a large, geographically diverse group of MSM. This allowed us, for example, to document that the reported intention to use CVCT did not have detectable differences by geography within the US. Last, our integration of the quantitative online approach with a parallel qualitative approach both allowed us to have better *a priori* concepts about motivations towards or against CVCT with which to code our pre-specified response options, and allowed us to triangulate our understanding of the survey results using qualitative data.

### Conclusions

We are in a re-emerging HIV epidemic among MSM [23], a group who is disproportionally underserved by available interventions. There is an urgent need for interventions for this group that is at greatest risk for HIV. Participants in our study expressed a high level of hypothetical acceptance of CVCT. When considered with available qualitative data [9], a picture emerges that CVCT may be most acceptable as a prevention tool for more committed partnerships and for younger men. Motivations for using CVCT may be a balanced mix of those around sexually transmitted disease prevention, and those around formalizing and strengthening relationships.

What is still unclear is whether the positive impact on reducing HIV transmission suggested in African studies will also accrue to male couples who participate in CVCT in the United States; the present study directly informed a randomized trial of an adapted intervention that was concluded in 2012. The scale-up, marketing, and evaluation of an adapted, US-based CVCT service for MSM should be guided by the important themes identified both in this survey, and in qualitative studies.

### Ethical Approval and Informed Consent

The present study was approved by the Emory University Institutional Review Board. Protocol number: 14085. Inform consent was received for all participants. Consent was documented electronically by asking the participant to check a box indicating consent in the online survey utility.
